# Clinical features and surgical treatment of magnetic bead ingestion in children: a single-center retrospective study

**DOI:** 10.3389/fped.2026.1869307

**Published:** 2026-06-29

**Authors:** Yuqiang Chen, Mao Ye, Zhen Zhang, Yandong Wei, Zhen Chen, Jingxuan Sun, Jianji Xu, Yijing Cheng, Xuelai Liu

**Affiliations:** 1 Department of General Surgery, Capital Center for Children’s Health, Capital Medical University, Capital Institute of Pediatrics, Beijing, China; 2 Department of Neonatal Surgery, Capital Center for Children’s Health, Capital Medical University, Capital Institute of Pediatrics, Beijing, China; 3 Child Health Big Data Research Center, Capital Center for Children’s Health, Capital Medical University, Capital Institute of Pediatrics, Beijing, China

**Keywords:** children, intestinal perforation, magnetic beads, retrospective study, surgical treatment

## Abstract

**Objective:**

To summarize the clinical features, complications, radiological characteristics, surgical management, and prognosis of magnetic bead ingestion (MBI) in children, and to propose a treatment-modality algorithm focused on selecting among endoscopic, laparoscopic, open, and combined surgical approaches.

**Method:**

A retrospective analysis was conducted on 24 pediatric MBI cases treated at the Capital Center for Children's Health, Capital Medical University, from January 2020 to October 2025. Data on demographics, ingestion details, clinical manifestations, laboratory and imaging findings, surgical procedures, intraoperative findings, and postoperative outcomes were analyzed.

**Results:**

The cohort comprised 15 boys and 9 girls (median age, 5.5 years; range, 1 year 1 month–11 years). Abdominal pain was the most common symptom (12 cases, 50.0%), followed by vomiting (9 cases, 37.5%); 10 children (41.7%) were asymptomatic. Among 14 children with intestinal perforation, elevated white blood cell count was observed in 8 (57.1%), elevated neutrophil percentage in 8 (57.1%), and elevated C-reactive protein in 6 (42.9%); notably, two perforation cases had all three markers within normal limits. Complications included intestinal adhesions (11 cases), perforation (14 cases), obstruction (3 cases), and necrosis (3 cases). Upright abdominal X-ray was the primary diagnostic modality (17 cases, 70.8%), followed by CT (4 cases, 16.7%) and ultrasound (2 cases, 8.3%). Eighteen children (75%) underwent surgery: laparoscopic exploration converted to open surgery (8 cases, 44.4%), converted to open surgery combined with gastroscopy (1 cases, 5.6%), completely laparoscopic surgery (5 cases, 27.8%), laparoscopic combined with gastroscopy (2 cases, 11.1%), and direct open surgery (2 cases, 11.1%); the remaining 6 cases experienced spontaneous bead expulsion.

**Conclusion:**

Magnetic bead ingestion in children can easily lead to severe intestinal injury. Clinical interventions should be individualized based on the location and number of foreign bodies as well as the presence of complications. Laparoscopic exploration is an important diagnostic and minimally invasive treatment approach, allowing flexible intraoperative decisions regarding conversion to open surgery or combination with endoscopy based on the actual situation. For children requiring surgical treatment, early intervention is key to improving prognosis.

## 1 Introduction

Children are naturally active and curious, making foreign body ingestion a common occurrence among them (1). Among such cases, magnetic beads—strongly magnetic toy components—have drawn increasing attention due to their potential to cause severe gastrointestinal injury after ingestion. When multiple magnetic beads are swallowed, they can attract one another across intestinal loops, trapping the intestinal wall between them and leading to ischemia, necrosis, or even perforation. This may result in intestinal obstruction and, in severe cases, can be life-threatening (2). In recent years, reports of magnetic bead ingestion (MBI) in children have been increasing both domestically and internationally; however, most are single-case reports (3). Systematic analyses focusing on clinical characteristics and surgical management strategies remain relatively sparse. This study retrospectively reviewed 24 cases of pediatric MBI treated at our center over the past six years, aiming to explore and summarize their clinical features, radiological diagnostic value, and surgical treatment strategies to provide a reference for clinical diagnosis and management. The present study focuses specifically on treatment-modality selection in pediatric magnetic bead ingestion. We aim to provide a practical framework for choosing between endoscopy, laparoscopy, laparotomy, and combined approaches based on foreign body location, symptom severity, and intraoperative findings.

## 2 Materials and methods

### 2.1 Study subjects

A total of 24 pediatric patients with magnetic bead ingestion (MBI) treated at both the General Surgery Department and the Neonatal Surgery Department in our center from January 2020 to October 2025 were included in this study. The studies involving humans were approved by the Ethics Committee of Capital Institute of Pediatrics. The ethical review number is: SHERLLM2026028.

### 2.2 Data collection methods

General information of the patients (including gender and age), time to medical consultation, number of beads ingested, clinical manifestations, radiological findings, surgical methods, intraoperative conditions, and postoperative examination data were collected from the hospital information system (HIS). The collected data were archived and retrospectively analyzed using Microsoft Excel.

### 2.3 Statistical analysis

Statistical analyses were performed using SPSS software (version 26.0; IBM Corp., Armonk, NY, USA). Categorical variables were summarized as frequencies and percentages. Owing to the small sample size and the presence of expected cell counts less than 5, Fisher's exact test was used to compare the association between delayed presentation (>24 h after ingestion) and intestinal perforation. A two-sided *P* value <0.05 was considered statistically significant.

## 3 Results

### 3.1 General information and clinical manifestations

Among the 24 children with magnetic bead ingestion (MBI), 15 were boys, and 9 were girls, with a median age of 5.5 years (range: 1 year and 1 month to 11 years). Of these, 23 cases were identified after the onset of symptoms such as abdominal pain or vomiting, or when caregivers noticed missing magnetic beads at home; 1 case was incidentally discovered during evaluation for an upper respiratory tract infection.

The time from ingestion to hospital presentation ranged from 5 h to 120 days. Thirteen children presented within 24 h of ingestion, 4 within 24–72 h, and 7 after more than 72 h. The number of beads ingested ranged from 2 to 48 (median: 7). The main clinical manifestations were abdominal pain (12 cases, 50.0%) and vomiting (9 cases, 37.5%) (Table 1).

**Table 1:** Summary of clinical data from 24 pediatric cases with magnetic beads ingestion in this cohort.

No.	Gender	Age (year)	Discovery prior to symptom onset	Presenting symptom	Ingestion-to-presentation interval	Quantity (beads)	Intraoperative bead location ≥2 sites	Complications	Examination (In-hospital)	Surgical approach	Postoperative outcome
1	Female	1	Yes	Vomiting	2 days	9	Yes	Intestinal Adhesion, Perforation, Obstruction	Ultrasound	Laparoscopic conversion to open surgery	Discharged 9 days post-operation
2	Male	8	Yes	Abdominal pain and vomiting	12 h	13	Yes	None	X-ray, Ultrasound	Laparoscopy combined with gastroscopy	Discharged 10 days post-operation
3	Female	9	Yes	None	1 day	7	–	None	–	Spontaneous expulsion	Discharged on the same day
4	Female	4	Yes	Abdominal pain and vomiting	1 day	7	No	Intestinal Adhesion, Perforation	X-ray	Laparoscopic conversion to open surgery	Discharged 8 days post-operation
5	Male	11	Yes	None	120 days	2	No	None	CT	laparoscopy	Discharged 6 days post-operation
6	Male	5	Yes	Abdominal pain	10 days	7	Yes	Intestinal Adhesion, Perforation	X-ray	Laparoscopy	Discharged 5 days post-operation
7	Female	8	Yes	None	12 h	12	No	None	X-ray, Ultrasound	Laparoscopy combined with gastroscopy	Discharged 6 days post-operation
8	Female	1	Yes	Vomiting	10 days	17	Yes	Intestinal Adhesion, Perforation	X-ray	Laparoscopic conversion to open surgery	Discharged 7 days post-operation
9	Male	7	No	Abdominal pain	7 days	32	Yes	Intestinal Adhesion, Perforation, Gastric perforation	CT	Conversion to open surgery after gastroscopic exploration and laparoscopic surgery	Discharged 10 days post-operation
10	Male	7	Yes	Abdominal pain and vomiting	4 days	3	Yes	Intestinal Adhesions, perforation, and necrosis	X-ray, Ultrasound	Laparoscopic conversion to open surgery	Discharged 11 days post-operation
11	Female	4	Yes	None	1 day	4	–	None	X-ray, Ultrasound	Spontaneous expulsion	Discharged on the same day
12	Male	9	Yes	Abdominal pain	1 day	6	No	None	CT	Laparoscopic conversion to open surgery	Discharged 5 days post-operation
13	Male	1	Yes	Abdominal pain	7 days	19	Yes	Intestinal Adhesions, perforation, and necrosis	X-ray, Ultrasound	laparoscopy	Discharged 14 days post-operation
14	Female	6	Yes	None	20 days	2	–	None	Ultrasound	Spontaneous expulsion	Discharged on the same day
15	Male	4	Yes	None	1 day	48	Yes	Intestinal Adhesions, perforation	CT	Laparoscopic conversion to open surgery	Discharged 9 days post-operation
16	Male	6	Yes	None	9 h	7	No	Intestinal Adhesions	X-ray	laparoscopy	Discharged 6 days post-operation
17	Male	8	Yes	None	20 h	4	–	Intestinal perforation	X-ray	Spontaneous expulsion	Discharged 1 day after hospital observation
18	Female	1	Yes	Abdominal pain and vomiting	12 h	13	Yes	Intestinal Adhesions, perforation	X-ray	Laparotomy	Discharged 8 days post-operation
19	Male	5	Yes	Abdominal pain and vomiting	3 days	18	Yes	Intestinal Perforation, Obstruction	X-ray	Laparotomy	Discharged 14 days post-operation
20	Male	10	Yes	None	5 h	2	No	None	X-ray	Spontaneous expulsion	Discharged on the same day
21	Male	1	Yes	Abdominal pain and vomiting	2 days	9	Yes	Intestinal Adhesion, Perforation, Obstruction	X-ray	laparoscopy	Discharged 13 days post-operation
22	Female	2	Yes	Abdominal pain	3 days	12	Yes	Intestinal perforation	X-ray, Ultrasound	Laparoscopic conversion to open surgery	Discharged 5 days post-operation
23	Male	8	Yes	None	8 h	6	–	None	X-ray	Spontaneous expulsion	Discharged on the same day
24	Male	1	Yes	Abdominal pain and vomiting	1 day	3	Yes	Intestinal perforation and necrosis	X-ray	Laparoscopic conversion to open surgery	Discharged 10 days post-operation

### 3.2 Hematological examination and imaging examinations

All 24 children in this cohort underwent preoperative hematological examination. Among the 14 children with intestinal perforation, the laboratory findings were as follows: an elevated white blood cell count (>10 × 10⁹/L) was observed in 8 children (57.1%), with a median of 12.1 × 10⁹/L (range, 6.7–18.2 × 10⁹/L); an elevated neutrophil percentage (>70%) was observed in 8 children (57.1%), with a median of 75.8% (range, 12.9%–96.0%); and an elevated C-reactive protein level (>8 mg/L) was observed in 6 children (42.9%), with a median of 7.5 mg/L (range, 0.5–67.9 mg/L). Notably, two children with perforation (Cases 8 and 9) had all three markers within normal limits, highlighting that unremarkable laboratory findings do not completely rule out intestinal perforation. Among the 10 children without perforation, only 2 (20.0%) had a mildly elevated white blood cell count, 1 (10.0%) had an elevated neutrophil percentage, and all had C-reactive protein levels within the normal range.

Of the 24 children, 1 child did not undergo imaging because the number and ingestion time of magnetic beads were clearly reported verbally. The remaining 23 children underwent imaging examinations at our center. Seventeen cases (70.8%) were diagnosed using upright abdominal X-ray, which clearly showed the number and shape of the beads; 2 cases (8.3%) were diagnosed by ultrasound because caregivers declined X-ray examination; and 4 cases (16.7%) were diagnosed by CT scan (Table 1).

Among the 24 cases, 11 children were referred from other hospitals, where relevant examinations had already been performed. In 4 cases, ultrasound failed to detect the magnetic beads, but upright abdominal X-ray confirmed the diagnosis. The remaining 7 cases were diagnosed directly by upright abdominal X-ray.

### 3.3 Treatment methods and intraoperative findings

18 of the 24 children underwent surgical treatment. The surgical approaches included: laparoscopic exploration converted to open surgery in 8 cases (44.4%), conversion to open surgery after gastroscopic exploration and laparoscopic surgery in 1 cases (5.6%), completely laparoscopic surgery in 5 cases (27.8%), laparoscopic combined with gastroscopic surgery in 2 cases (11.1%), and open surgery in 2 cases (11.1%). 6 children (25.0%) were successfully managed conservatively, with spontaneous expulsion of the beads (Table 1).

In the 18 surgically treated cases, intraoperative exploration revealed that the magnetic beads were mainly located in the jejunum and ileum (15 cases), while a few (3 cases) had beads simultaneously in the stomach and colon. In most cases, the magnetic beads were aggregated rather than isolated, forming clusters that spanned multiple bowel loops, creating “bead-string” or “bracelet-like” configurations, consistent with preoperative imaging findings (Table 1).

### 3.4 Analysis of complications caused by magnetic beads

Among the 24 cases, 15 (62.5%) had complications confirmed either by preoperative imaging or intraoperative findings. These included intestinal perforation in 14 cases, intestinal obstruction in 3 cases, intestinal necrosis in 3 cases, and intestinal adhesions in 11 cases. In 9 cases (37.5%), no severe complications were identified (Table 1).

### 3.5 Postoperative recovery

All children recovered uneventfully after surgery and were discharged. The postoperative hospital stay ranged from 5 to 14 days (median, 8.5 days). The follow-up protocol comprised an outpatient review with a full blood count and upright abdominal radiography at 1 week after discharge, and repeat upright abdominal radiography at 1 month and 6 months postoperatively. All children were followed up for 6 months. No complications such as abdominal pain, vomiting, or intestinal obstruction were observed during follow-up.

## 4 Discussion

Magnetic bead ingestion (MBI) has emerged as an increasingly concerning type of pediatric accidental injury worldwide in recent years. Its clinical manifestations vary widely, ranging from asymptomatic to acute abdomen presentations (1). Common symptoms include abdominal pain, vomiting, and fever (1, 4); however, a considerable proportion of patients (approximately 43%) may remain asymptomatic or show only nonspecific symptoms when the number of beads ingested is small or the time since ingestion is short, making diagnosis challenging (5). A clear ingestion history is an important basis for diagnosing MBI, but imaging examinations also play a crucial diagnostic role (6, 7).

Because of their strong magnetic properties, multiple beads within the gastrointestinal tract tend to attract each other, compressing the intestinal wall and leading to ischemia, perforation, necrosis, or even the formation of multiple enteric fistulas (3). This inter-bead attraction is the key mechanism that causes pressure necrosis and perforation of adjacent intestinal loops. This study systematically analyzed 24 pediatric MBI cases treated at our hospital over the past six years to characterize their clinical features, risk factors, and treatment strategies, thereby providing evidence to guide standardized clinical management.

**Figure 1:**
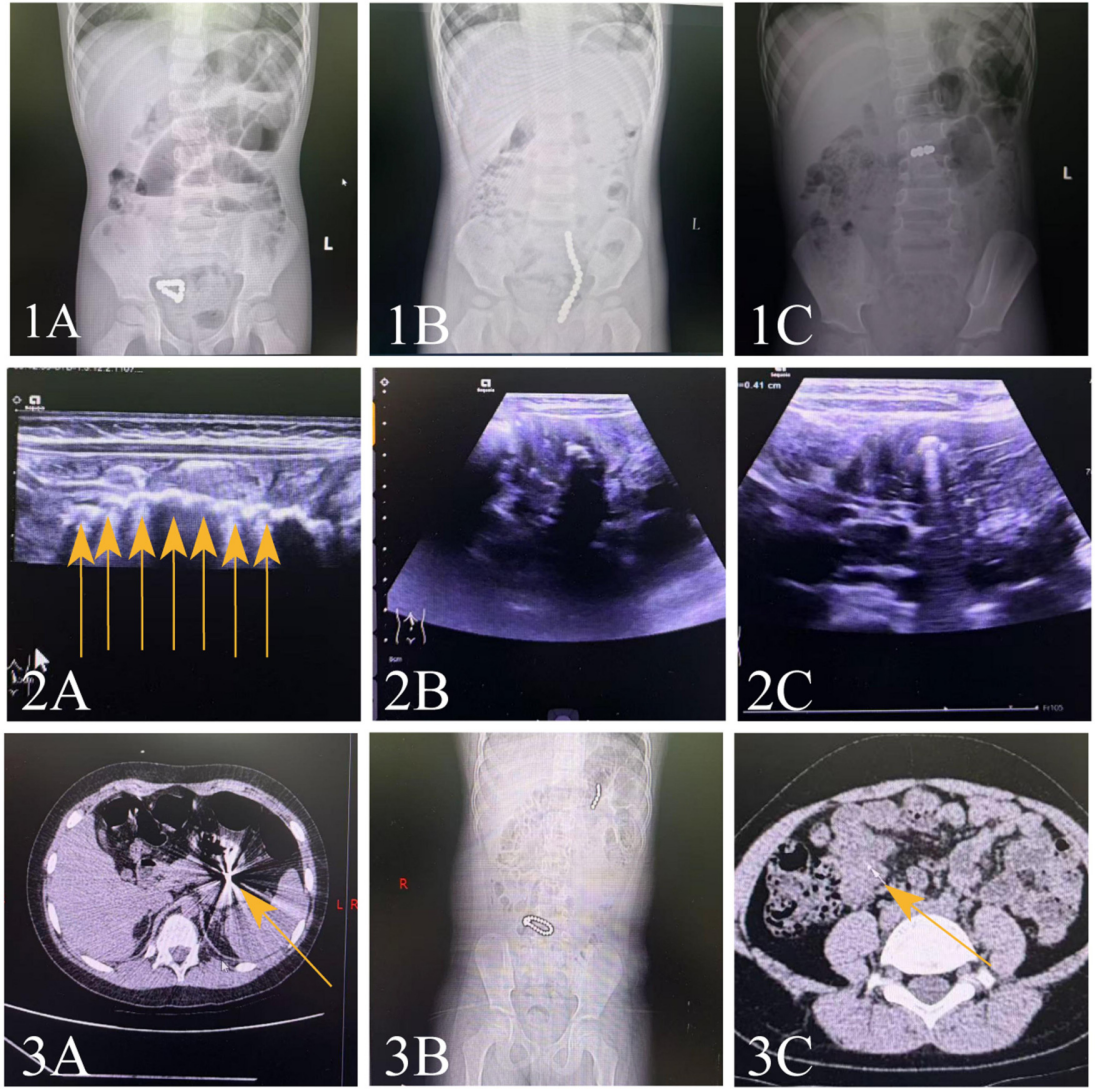
Using different imaging modalities to evaluate the preoperative location and condition of magnetic beads within the abdominal cavity. **(1A–1C)** show upright abdominal radiographs demonstrating clearly visible magnetic beads distributed within the gastrointestinal tract, presenting as “bracelet-like” and “string-of-beads–like” configurations. **(2A–2C)** show the ultrasound findings of one child. Spherical hyperechoic foci with posterior acoustic shadowing are visible within the gastric lumen. More than ten bead-like hyperechoic foci are arranged in a row within the mid-upper abdominal bowel loops, accompanied by focal bowel wall thickening (yellow arrow). A whirlpool-like torsion is observed at the root of the upper abdominal mesentery. **(3A,3B)** show CT findings of another child. In **(3A)**, hyperdense foreign bodies are visible within the lower abdominal bowel loops, accompanied by marked metal artifacts (yellow arrow). **(3B)**, an X-ray image reconstructed from CT, demonstrates the distinct distribution of magnetic beads in varying quantities. **(3C)** shows CT findings of a third child, again revealing hyperdense foreign bodies within the lower abdominal bowel loops with prominent metal artifacts (yellow arrow).

Our findings revealed the following: ① MBI predominantly affected preschool and school-aged boys (median age 5.5 years), consistent with both domestic and international reports (1); ② The number of ingested magnetic beads appeared to be associated with the development of complications. Among the 9 children who ingested ≥10 beads, 7 developed intestinal perforation, yielding a perforation rate of 77.8% (7/9), whereas among the 15 children who ingested <10 beads, 7 (46.7%) developed perforation. This finding suggests that a greater number of beads increases the extent and intensity of intestinal wall compression, suggesting a potential association with a higher risk of perforation; ③ Regarding the relationship between time to presentation and complications, the intestinal perforation rate was higher among children presenting >24 h after ingestion than among those presenting within 24 h. Intestinal perforation was observed in 9 of 11 children who presented >24 h after ingestion and in 5 of 13 children who presented within 24 h. Fisher's exact test showed a statistically significant association between delayed presentation and intestinal perforation (two-sided *P* = 0.047). Nevertheless, from a clinical perspective, delayed presentation remains an important concern. Sustained magnetic attraction between beads within the gastrointestinal tract may progressively aggravate local ischemia and inflammatory injury, thereby increasing the risk of severe complications such as perforation, necrosis, and intestinal obstruction. Therefore, early recognition and prompt intervention remain essential for improving clinical outcomes in children with magnetic bead ingestion. ④ Imaging examinations play an indispensable role in diagnosis and complication assessment. Upright abdominal X-ray remains the first-line investigation, clearly revealing the high-density magnetic foreign bodies and their distribution within the gastrointestinal tract (8). When intestinal perforation, adhesion, or obstruction is suspected, CT provides higher sensitivity (8). In our cohort, upright abdominal X-ray demonstrated high diagnostic accuracy, effectively identifying the approximate position, number, and pattern of beads. Ultrasonography also has complementary value - it can assess bowel wall thickening, peritoneal effusion, and peristalsis, and is advantageous in evaluating bowel wall perfusion and early perforation. However, it is highly operator-dependent and less reliable for precise bead localization. In resource-limited settings, upright abdominal X-ray should be the preferred diagnostic method; ⑤ Although CT is not the first choice, it is valuable in complex cases (e.g., with intestinal adhesions, obstruction, or intra-abdominal abscess), providing comprehensive information about the bowel and surrounding tissues to guide surgical planning. However, radiologists are often unable to distinguish the exact location of foreign bodies (especially when determining if an object is in the small versus large bowel), we advocate selective CT examination to determine the location of magnetic bead foreign bodies. However, in clinical practice, the decision is made flexibly, and not every patient undergoes CT examination. Factors such as the interval between magnetic bead ingestion and hospital admission, the patient's symptoms and physical signs at presentation, together with findings from abdominal plain radiography (KUB), can also help determine the location of the magnetic beads. For example, when the ingestion occurred only a short time earlier (1–2 h), the patient has no symptoms of perforation or abdominal pain, KUB demonstrates that the magnetic beads are located in the stomach or duodenum, and no free subdiaphragmatic air is detected, CT examination may be considered unnecessary (Figure 1).

**Figure 2:**
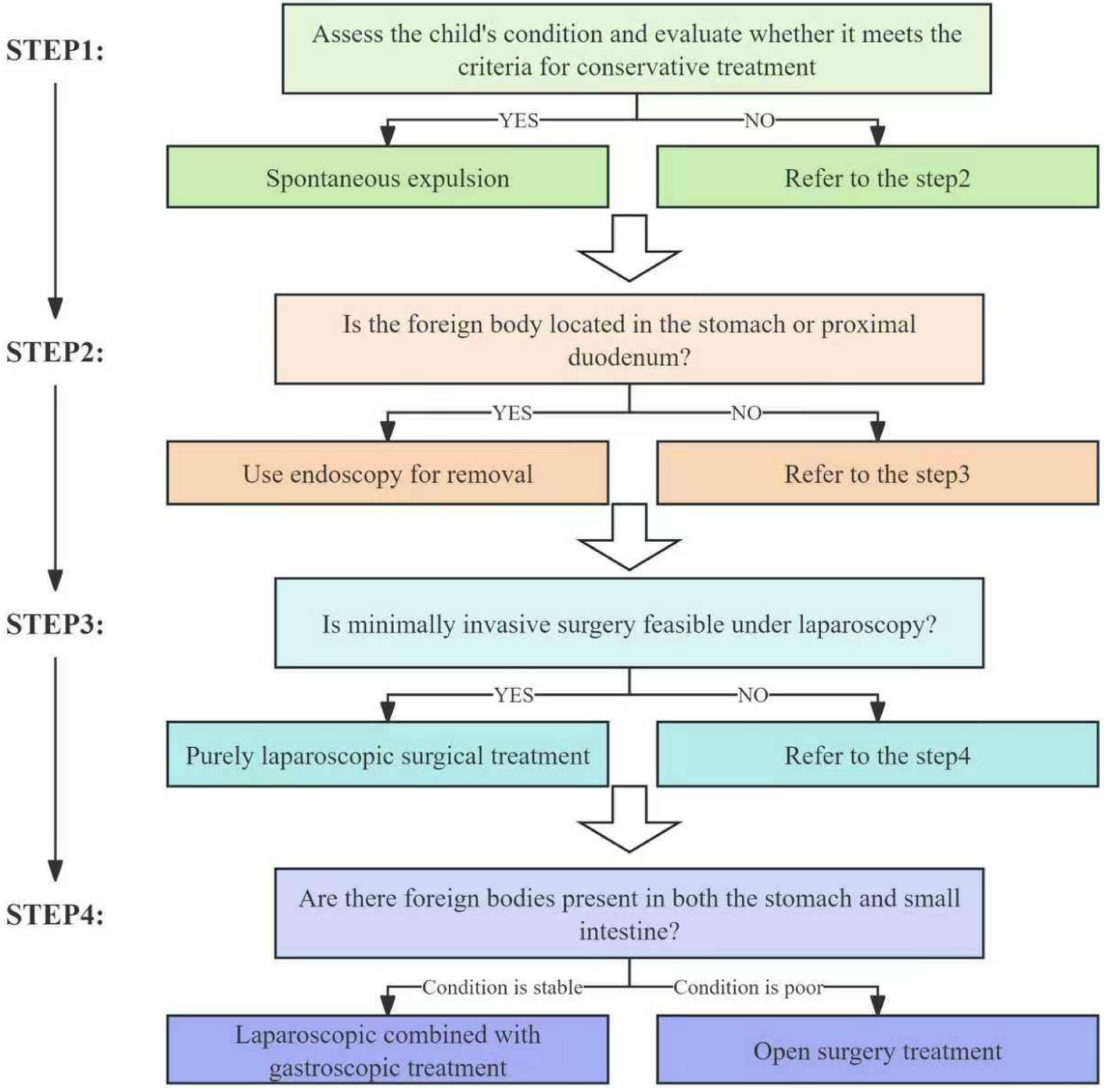
Individualized treatment-modality selection pathway. The flowchart outlines a stepwise approach to determining the optimal treatment strategy for pediatric patients who have ingested magnetic beads. The process begins with an initial assessment of the patient's eligibility for conservative management (Step 1). If intervention is required, the flowchart subsequently guides the choice of extraction technique based on the foreign body's location (endoscopy for gastroduodenal objects, Step 2) and the feasibility of minimally invasive surgery (laparoscopy, Step 3). The final step (Step 4) addresses complex scenarios, recommending a combined laparoscopic-gastroscopic approach for stable patients with objects in both the stomach and small intestine, or open surgery for unstable patients. This flowchart serves to standardize clinical decision-making and prioritize minimally invasive techniques when feasible.

Management of MBI emphasizes individualization and timeliness. It places greater emphasis on dynamic intraoperative decision-making and individualized minimally invasive strategy selection, with treatment strategies tailored according to the patient's symptoms, the number and distribution of magnetic beads, and the presence of complications. The treatment approach depends on the patient's symptoms, the number and distribution of magnetic beads, and the presence of complications. In our cohort, 75% of patients required surgical intervention, highlighting the frequent need for operative management. Based on our experience, we propose the following individualized treatment pathway (Figure 2): ① conservative management: For asymptomatic or mildly symptomatic patients in whom the beads have passed beyond the pylorus, short-term conservative observation can be considered (9). However, because prolonged retention increases complication risk (2), close clinical and radiologic monitoring is essential until all beads have passed. In our experience, the decision to observe should not be based on bead number alone. Rather, it should be guided by the patient's symptoms, the presence of bead aggregation, progression on serial imaging, and any evidence of bowel compression or injury. Therefore, the proposed threshold of a very limited number of beads was intended only as a practical reference derived from institutional experience, not as an absolute cutoff. For example, Patient #11, who ingested four beads, remained asymptomatic and expelled the beads spontaneously, whereas Patient #24 developed intestinal perforation despite ingesting only three beads, because the beads caused persistent magnetic attraction and bowel injury. This may be due to the inconsistent timing of ingestion of individual magnetic beads, resulting in their distribution across different intestinal loops where they attracted each other. These cases underscore the need for individualized decision-making; In this regard, our experience suggests that serial abdominal radiography (serial KUB) follow-up is essential. We recommend hospital admission for serial KUB monitoring, with imaging performed every 6–8 h to assess changes in the location of the magnetic foreign bodies, and discharge should only be considered after complete passage of all magnetic beads. In addition, we advocate spontaneous passage under normal gastrointestinal peristalsis rather than the use of laxatives to facilitate bead expulsion. The rationale is that laxatives may accelerate gastrointestinal mucosal secretion and edema, thereby increasing bowel wall fragility. Once magnetic attraction between beads causes local ischemia of the intestinal wall, the use of laxatives may further increase the risk of bowel wall injury and perforation. ② Endoscopic removal: Gastroscopy is the preferred approach for retrieving magnetic foreign bodies in the esophagus or stomach (9). Compared with traditional surgery, endoscopic retrieval offers higher efficiency and success rates, with no reported adverse events (10). It is appropriate for beads located in the stomach or proximal duodenum without signs of perforation; ③ Laparoscopy: With its minimally invasive advantages, laparoscopy has become the preferred surgical option for MBI (11). It allows diagnostic exploration, foreign body matter retrieval, and perforation repair, particularly for small intestinal lesions. This approach is suitable for the majority of MBI cases in which the magnetic beads are located in the small intestine. However, in children with MBI, the likelihood of conversion to open surgery is high when intraoperative findings confirm extensive intestinal adhesions or severe peritoneal contamination resulting from bead-induced perforation (12). The decision to convert should be guided by an intraoperative assessment of the number of perforations, the severity of intestinal adhesions, the degree of bowel distension, and the surgeon's own proficiency with laparoscopic techniques. ④Conversion to laparotomy: For complex cases involving multiple perforations, extensive intestinal adhesions or severe necrosis, conversion to open surgery is a prudent choice, enabling comprehensive exploration and management of diseased bowel segments (1, 12). Conversion should be viewed as a safety strategy rather than a procedural failure. The conversion-to-open-surgery rate in this cohort was 50.0% (9/18), which was primarily attributable to severe intestinal adhesions (6 cases), multiple perforations (8 cases), and densely adherent bowel loops caused by inter-bead magnetic attraction (5 cases), with multiple factors coexisting in several cases. All 11 children who underwent conversion to open surgery or primary open surgery had an uneventful postoperative recovery, with a median hospital stay of 9.0 days (range, 5–14 days). No complications—including wound infection or anastomotic leakage—were observed, and no long-term complications were identified during the 6-month follow-up. These findings indicate that, although conversion to open surgery entails additional surgical trauma, it is a safe and reliable strategy and serves as an important salvage measure to ensure patient safety.

Furthermore, combined laparoscopic-endoscopic surgery represents another effective treatment strategy. For cases in which magnetic beads are simultaneously present in the stomach and the small intestine (or colon), a combined laparoscopic-gastroscopic approach can improve retrieval efficiency while minimizing surgical trauma (3). In this cohort, two cases treated with combined laparoscopic-gastroscopic surgery substantiates the value of the aforementioned individualized treatment pathway in complex scenarios. The patient had ingested a total of 13 magnetic beads in two separate episodes. The distinctiveness of this case lay in the final location of the beads—lodged in the descending duodenum and the hepatic flexure of the colon, respectively—forming an unusual configuration of tightly coupled magnetic attraction spanning the upper and lower gastrointestinal tract. This posed a considerable challenge to treatment decision-making: gastroscopy alone would have been unable to address the colonic beads; laparoscopic surgery alone would have required separate enterotomies through both the duodenal and colonic walls to retrieve the clustered beads, entailing substantial trauma; and open surgery would have forfeited the advantages of a minimally invasive approach. Adhering to the principles of individualized and minimally invasive treatment, we performed a combined laparoscopic-gastroscopic procedure. Intraoperatively, the primary role of laparoscopy was to provide an accurate diagnosis by directly confirming that the duodenal and colonic walls, although tightly apposed by magnetic attraction, had not yet perforated—a finding that obviated the need for more traumatic procedures such as intestinal resection and anastomosis. Subsequently, a coordinated strategy was employed: the laparoscopic team first retrieved the 2 colonic beads via a small colotomy and repaired the colonic wall, after which the endoscopist entered the duodenum and retrieved the remaining 11 beads. The advantages of this approach were threefold. First, trauma minimization: by limiting the number of enterotomies, surgical trauma and the risk of postoperative complications were reduced. Second, maximized efficiency: synchronous dual-endoscopic operation allowed both sites of foreign body impaction to be addressed in a single procedure, thereby shortening the operative time. Third, integration of diagnosis and treatment: laparoscopy enabled comprehensive assessment of the abdominal cavity, ensuring that no perforation at other sites was missed. This case further demonstrates that, for complex magnetic foreign bodies located in different gastrointestinal segments—particularly those forming transmural magnetic attraction across the upper and lower digestive tract—the combined dual-endoscopic approach is not merely a technical addition, but rather an optimized strategic integration grounded in precise assessment. Close imaging surveillance of the foreign body dynamics before surgery, combined with flexible intraoperative decision-making based on real-time laparoscopic feedback, is key to the successful implementation of individualized and precision treatment.

When multiple beads are found intraoperatively forming “dumbbell-shaped” compression necrosis or entero-enteric fistulas, thorough bowel exploration is essential to avoid missing small perforations or segmental necrosis. For viable bowel, enterotomy with bead removal is appropriate; for necrotic or perforated segments, bowel resection and anastomosis should be performed. In this cohort, the intestinal perforation rate among children with MBI who presented with abdominal pain or vomiting was 85.7% (12/14), markedly higher than the 20.0% (2/10) observed in asymptomatic children, a finding consistent with the literature (9), most commonly in the small intestine (12). Patients who successfully underwent laparoscopic surgery tended to have shorter hospital stays than those requiring conversion to open surgery, underscoring the recovery benefits of minimally invasive techniques (12, 13).

In summary, MBI is a potentially life-threatening emergency in children that readily leads to serious complications such as intestinal perforation. Imaging examinations—preferably upright abdominal radiography combined with ultrasonography—can establish the diagnosis preoperatively. The clinical management of MBI should follow an individualized principle, encompassing a comprehensive, stepwise diagnostic and therapeutic framework that includes conservative observation, endoscopy, and surgical intervention (laparoscopic or open surgery). Once the intraoperative location of the magnetic beads is confirmed, the appropriate treatment modality can be selected according to the specific site of impaction. Surgical intervention is mandatory when intestinal perforation is present; laparoscopic exploration serves both diagnostic and therapeutic roles, although conversion to open surgery is required in the majority of severe cases. In this cohort, all children recovered uneventfully without complications, underscoring the importance of individualized surgical decision-making and meticulous operative technique. Nonetheless, this study is a single-center retrospective analysis with a limited sample size. Future large-scale, multicenter, cross-regional prospective studies are warranted to further elucidate the impact of various MBI scenarios on tissue injury, and to develop more precise imaging-based predictive models and indicators, thereby establishing a more streamlined and optimized treatment pathway. Future prospective multicenter studies with larger sample sizes are also needed to validate the proposed algorithm and to further evaluate its performance, including false-positive and false-negative rates.

## Data Availability

The original contributions presented in the study are included in the article/Supplementary Material, further inquiries can be directed to the corresponding author.
